# Sodium-Dependent Glucose Transporter 1 (SGLT1) Stabled by HER2 Promotes Breast Cancer Cell Proliferation by Activation of the PI3K/Akt/mTOR Signaling Pathway in HER2+ Breast Cancer

**DOI:** 10.1155/2020/6103542

**Published:** 2020-04-21

**Authors:** Jinlu Wang, Hongfei Ji, Xingjian Niu, Lei Yin, Yiran Wang, Yucui Gu, Dongbo Li, Han Zhang, Minghui Lu, Fengxia Zhang, Qingyuan Zhang

**Affiliations:** ^1^Department of Medical Oncology, Harbin Medical University Cancer Hospital, Harbin Medical University, Harbin, 150081 Heilongjiang, China; ^2^Institute of Cancer Prevention and Treatment, Harbin Medical University, Harbin, 150081 Heilongjiang, China; ^3^Heilongjiang Academy of Medical Sciences, Harbin, 150081 Heilongjiang, China; ^4^Clinical Laboratory, The People's Hospital of Daqing, Daqing, 163316 Heilongjiang, China

## Abstract

Aerobic glycolysis is a hallmark of tumor cells. SGLT1 plays a vital role in glucose metabolism. However, whether SGLT1 could promote cell growth and proliferation in breast cancer remains unclear. Here, we investigated the expression of SGLT1 in breast cancer and examined its role in malignant behavior and prognosis. Further, we examined the SGLT1 expression in breast cancer tissues and its relationship with clinicopathologic characteristics. We clarified that SGLT1 was overexpressed in HER2+ breast cancer cell lines and was affected by HER2 status. We further found that SGLT1 affected breast cancer cell proliferation and patient survival by mediating cell survival pathway activation. SGLT1 was overexpressed in HER2+ breast cancers and associated with lymph node metastasis and HER2+ status. Inhibition of HER2 decreased SGLT1 expression, and the extracellular acidification rate was also reduced in the UACC812 and SKBR3 cell lines. These changes could be reversed by proteasome inhibitor treatment. Knockdown of SGLT1 blocked PI3K/Akt/mTOR signaling, thereby inhibiting cell proliferation. Further, we demonstrated that high SGLT1 was significantly correlated with shorter survival in all breast cancer patients and specifically in HER2+ breast cancer patients. Therefore, we conclude that SGLT1 is overexpressed in HER2+ breast cancer, thereby promoting cell proliferation and shortening survival by activating PI3K/Akt/mTOR signaling. This study submits that SGLT1 is promising not only as a novel biomarker of HER2+ breast cancer subtype but also as a potential drug target.

## 1. Introduction

Breast cancer is the second leading cause of cancer-related death in women worldwide [1–3], and its incidence has risen rapidly in recent years [4, 5]. Although significant improvements in DFS and OS have been achieved by comprehensive adjuvant therapy [6], breast cancer patients diagnosed at advanced stages still have poor prognosis [7]. The HER2+ subtype accounts for 15-20% of breast cancer cases and is prone to recurrence and metastasis [8, 9]. Several anti-HER2 monoclonal antibodies and receptor tyrosine kinase inhibitors have been approved by the FDA [10]. However, de novo and acquired resistance [11] to drugs targeting HER2 are common, and the resultant refractory disease can seriously affect prognosis. Therefore, how to enhance the response to therapeutic drugs and improve survival is still a subject of extensive research.

In recent years, it has become clear that a series of metabolic alterations are initiating factors in tumorigenesis [12, 13]. Metabolic alterations may even take precedence over morphological changes in breast cancer, among which aerobic glycolysis in cancer cells plays a pivotal role [14]. Malignant cells have accelerated glucose uptake and utilization compared to their normal counterparts [15, 16]. Glucose transport proteins are used for glucose uptake to allow for a high rate of glycolysis under hypoxia to promote survival and drug resistance. Two kinds of glucose transport proteins have been identified to play a role in human cancers [16, 17]. One is the facilitative glucose transporters, which harness the extra-/intracellular glucose differential to passively transport glucose. The second kind, SGLTs, mediates active transport, utilizing the concentration gradient of electrochemical sodium ions across the cell membrane to transport glucose [18], regardless of the extracellular glucose concentration. In human cells, there are two main SGLTs, SGLT1 and SGLT2 [19], with different physiological functions. SGLT1 is the major active isoform. Studies have found that high levels of SGLT1 are associated with poor survival in various epithelial cancers, including pancreatic cancer [20], ovarian cancer [21], cervical cancer [22], colorectal cancer [23], prostate cancer, and renal cell cancer [24]. According to a study by Lai et al., SGLT1 can act as an independent unfavorable prognostic marker for ovarian cancer [21], whereas another group found that high SGLT1 expression in pancreatic cancer was significantly associated with longer DFS in younger patients [25]. To date, however, the expression of SGLT1 in breast cancer has not been explored, and the role of SGLT1 is still unclear. Therefore, studies focusing on the expression of SGLT1 and its effect on abnormal glucose metabolism in breast cancer cells are needed.

HER2 belongs to the HER family, which also includes EGFR, HER3, and HER4 [26]. HER2 is an oncogenic protein whose amplification has been confirmed to play important roles in the development and progression of breast cancer [27]. Global clinical trials are under way to evaluate novel anti-HER2 antibodies and small molecules targeting its tyrosine kinase activity [28–30]. Accumulating evidence suggests that the HER family member EGFR has both kinase-dependent and kinase-independent functions. It can interact with SGLT through its kinase-independent function to promote the survival of tumor cells. In addition, Janku et al. also found that IGF-1R interacted with SGLT1 in a manner similar to that previously reported for EGFR [31]. Considering that both HER2 and EGFR belong to the HER receptor tyrosine kinase family and perform similar functions in cells, it is plausible that this kinase-independent function is not unique to EGFR and IGF-1R but might also occur in other receptor tyrosine kinases, such as HER2. Moreover, some literatures have reported that SGLT1 is also involved in the activation of some downstream signaling pathways, thus affecting the course of some diseases [32, 33]. For example, ChingYing et al. demonstrated that SGLT1 reduced epithelial barrier damage and bacterial translocation by activating PI3K/Akt/mTOR signaling in intestinal ischemia. Therefore, it is important to verify whether SGLT1 and HER2 can interact and activate downstream signaling cascades that are involved in orchestrating the proliferation and survival of tumor cells, thus fostering tumor progression.

Hence, the aims of this present study are to (1) determine the expression of SGLT1in different molecular subtypes of breast cancer by IHC and its association with clinicopathologic features, (2) determine how the expression of SGLT1 is affected by the HER2 status by western blot and assess the effect of SGLT1 on glycolysis in HER2+ breast cancer cell lines, and (3) investigate the role of SGLT1 in cell proliferation and patient survival via downstream signaling pathways.

## 2. Materials and Methods

### 2.1. Patient Selection

A total of 216 primary breast cancer patients diagnosed at the Third Affiliated Hospital, Harbin Medical University, China, between January 2006 and December 2012 were enrolled. Among the 216 invasive breast cancer samples with complete information for molecular subtypes, 93 (43.1%) samples were luminal-like subtype, 81 (37.5%) samples were HER2+ subtype, and 42 (19.4%) samples were basal-like subtype. Paraffin-embedded archival pathologic samples were obtained prior to chemotherapy and/or radiation therapy. Patient information and clinicopathologic features, including age, tumor size, lymph node involvement, menopause status, and ER, PR, and HER2 status, were recorded and assessed for the whole study cohort in a standardized manner. The median follow-up was 48.5 months (range, 10-80 months). The study followed the tenets of the Declaration of Helsinki and was approved by the medical ethics committee of the Third Affiliated Hospital of Harbin Medical University. The patients provided their written informed consent to participate in the study, and all samples were obtained after informed consent was provided.

### 2.2. Cell Cultures and Treatments

The HER2+ breast cancer cell lines UACC812 and SKBR3 were originally purchased from the American Type Culture Collection (Manassas, VA, USA) and authenticated by short tandem repeat analysis. UACC812 and SKBR3 cells were cultured in DMEM and RPMI-1640, respectively, supplemented with 10% fetal bovine serum and 1% penicillin/streptomycin. All cells were cultured in a humidified incubator containing 5% CO_2_ at 37°C. For experiments, cells were treated with trastuzumab (20 *μ*M), pertuzumab (20 *μ*M), phlorizin (100 *μ*M), MG132 (10 *μ*M), or vehicle. The promoter-driven siRNA vector with GFP expression was constructed by GenScript Corp. The target sequence for SGLT1 siRNA was TCTTCCGCATCCAGGTCAAT, and the control siRNA sequence was GAACAATGTTGACCAGGTGA.

### 2.3. IHC

Immunohistochemical staining was performed using a standard streptavidin-biotin-peroxidase complex method. Briefly, 4 *μ*m thick slides were deparaffinized and subjected to heat-induced epitope retrieval before incubation with SGLT1 antibody. For this purpose, slides were immersed in sodium citrate buffer at pH 6.0 and heated in a high-pressure cooker for 1.5 min. After blocking with 3% hydrogen peroxide for 30 min at 37°C, the slides were incubated at 4°C overnight with primary anti-SGLT1 antibody (Abcam) at a dilution of 1 : 300. Afterwards, the slides were stained with the secondary rabbit antibody. After visualization of the reaction with the DAB chromogen for 3 min, the slides were counterstained with hematoxylin and covered with a glycerin gel. The negative staining result was generated by replacing SGLT1 with PBS plus 1% bovine serum albumin.

IHC staining results were scored by two experienced independent investigators who provided a consensus opinion of stain patterns by light microscopy. The cytoplasmic immunoreactivity of SGLT1 protein was scored in a semiquantitative method as follows. The sections were divided into those with negative and positive expression. The percentage of positive staining was classified as 0 (<10%), 1 (10-30%), 2 (30-50%), or 3 (>50%), and the staining intensity was defined as 0 (absent), 1 (weak), 2 (moderate), or 3 (strong). Sections with a total score (percentage score multiplied by intensity score) greater than 2 were regarded as SGLT1-positive; all other sections were considered SGLT1-negative.

### 2.4. Western Blot

Briefly, cells were lysed with RIPA buffer and total protein was extracted. Protein concentrations were measured using the BCA protein assay kit (Thermo Fisher Scientific) according to the manufacturer's instructions. Equal amounts of the whole protein extracts pooled from triplicate samples separated by SDS-PAGE gels were transferred to PVDF membranes, followed by blocking with 10% skim milk for 1 h and then incubation with primary antibodies at optimized concentrations overnight at 4°C. The PVDF membranes were washed with 0.1% TBST three times for 5 min each before incubation with the corresponding secondary antibody conjugated to horseradish peroxidase (1 : 10,000 dilution) for 1 h at room temperature. After a 30 min wash, specific binding was visualized by an enhanced chemiluminescence system. The results are presented as the protein level fold changes normalized to *β*-actin. These experiments were repeated at least three times.

### 2.5. Measurement of the ECAR

The ECAR was measured using the Seahorse XF Glycolysis Stress Test Kit and the Seahorse XF96 Extracellular Flux Analyzer (Seahorse Bioscience). Briefly, 2 × 10^4^ UACC812 and SKBR3 cells/well were seeded into a Seahorse XF96 cell culture microplate followed by overnight incubation. After baseline measurements for ECAR, cells were treated as stated above for 24 h. The ECAR values were calculated by normalizing the cell number.

### 2.6. Cell Transfections

Briefly, 2 × 10^6^ cells/mL were seeded in a 6-well plate. When the cell density reached approximately 70% in conventional cell culture, SGLT1 siRNA or control siRNA was transfected into cells using Lipofectamine 2000 (Invitrogen) according to the manufacturer's instructions. After 4 h, the medium was replaced, and cells were incubated in a humidified incubator for another 48 h. Following transfection, cell lysates were analyzed by western blot.

### 2.7. Cell Growth Assay

Cell growth was determined using the Cell Counting Kit-8 (CCK-8) assay in 96-well plates. A total of 5,000 cells suspended in 200 *μ*L normal medium were seeded in each well. On the second day, the medium was replaced with fresh medium and treated as stated above. Cell proliferation was measured at different times (0, 12, 24, 36, and 48 h) using a Microplate Reader (Bio-Rad) after 10 *μ*L CCK-8 reagent (Beyotime Biotechnology) was added to each well and incubated for 4 h at 37°C. Each experiment was done in triplicate.

### 2.8. Statistical Analysis

The data were subjected to statistical analysis using GraphPad Prism 6 and SPSS 20.0 software for Windows. A chi-squared test was used to evaluate the association of SGLT1 expression and clinicopathologic parameters in breast cancer patients. Comparison of two groups was statistically calculated by Student's *t*-test. DFS was defined as the period from the initial diagnosis to disease progression, death, or the end of follow-up. OS was estimated as the period from the initial diagnosis to death or the end of follow-up. The Kaplan-Meier method was used to estimate DFS and OS. The data were presented as the mean values ± SDs. *p* values less than 0.05 were defined as statistically significant.

## 3. Results

### 3.1. SGLT1 Expression in Breast Cancer and Its Association with Clinicopathologic Features

**Figure 1:**
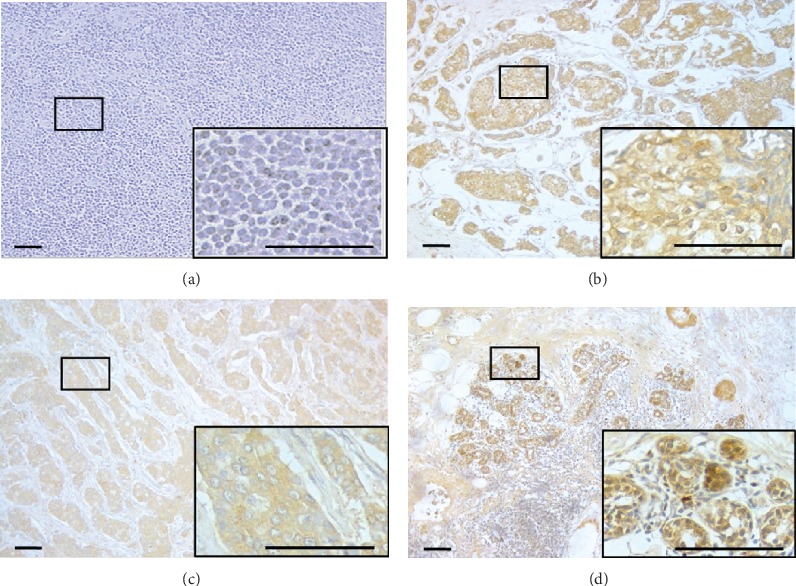
Representative staining of SGLT1 in different subtypes of breast cancer (×100, ×400). (a) Negative immunoreactions. Scale bar: 100 *μ*m. (b) Positive staining of SGLT1 in luminal-like breast cancer. (c) Positive staining of SGLT1 in HER2+ breast cancer. (d) Positive staining of SGLT1 in basal-like breast cancer.

The basic details of clinicopathologic characteristics in this cohort are listed in Table 1. SGLT1 exhibited diffuse staining in the membrane and cytoplasm. Representative IHC staining is shown in Figure 1. SGLT1 was highly expressed in HER2+ breast cancer, with a typical strong positive staining. Almost half (43.2%) of samples were SGLT1-positive. The associations between SGLT1 expression in breast cancer and clinicopathologic parameters were further analyzed. High SGLT1 expression was significantly correlated with lymph node metastasis (*p* = 0.004) and HER2+ status (*p* = 0.049) in breast cancer patients. There was no correlation with age, tumor size, menopause status, or ER or PR status (Table 1).

### 3.2. HER2 Inhibition Decreases SGLT1 Expression in HER2+ Breast Cancer Lines

**Figure 2:**
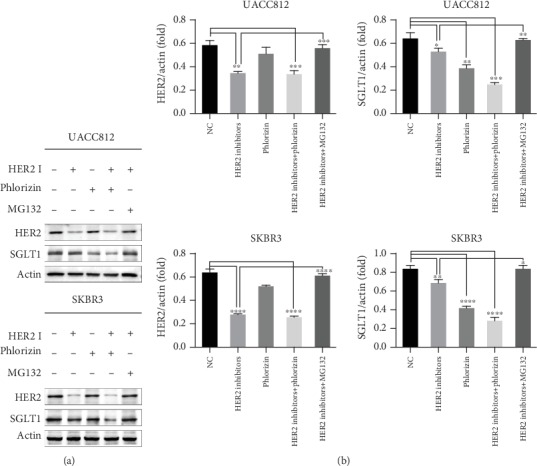
Representative western blot of HER2 and SGLT1 expression levels and quantification relative to actin in UACC812 and SKBR3 cells. UACC812 and SKBR3 cells were treated with HER2 inhibitors (HER2 I), Phlorizin, MG132 alone, or in combination for 48 h. Untreated cells were used as a negative control (NC). (a) A representative WB detection result of three independent experiments was shown. (b) The relative levels of HER2 and SGLT1 protein in UACC812 and SKBR3 cells. Statistically significant differences between groups were displayed; ^∗∗∗∗^*p* < 0.0001; ^∗∗∗^*p* < 0.001; ^∗∗^*p* < 0.01; ^∗^*p* < 0.05.

We confirmed that SGLT1 was overexpressed in HER2+ breast cancer tissues and associated with HER2 status. Therefore, we tested the expression of SGLT1 based on HER2 activity in vitro by western blot using the UACC812 and SKBR3 cell lines. *β*-Actin served as a loading control. Both UACC812 and SKBR3 cells were treated with HER2 inhibitors (trastuzumab and pertuzumab), phlorizin (a competitive inhibitor of SGLT1), or HER2 inhibitors plus phlorizin. UACC812 and SKBR3 cells without any treatment were employed as controls. As shown in Figure 2, the levels of HER2 decreased after trastuzumab and pertuzumab treatment in these two cell lines. Compared with the control cells, SGLT1 expression decreased after treatment with HER2 inhibitors, whereas that in cells treated with phlorizin and HER2 inhibitors plus phlorizin decreased more significantly. Therefore, we concluded that HER2 sustains the expression of SGLT1, although other factors may be playing a role.

### 3.3. The ECAR Decreases with HER2 Inhibition

**Figure 3:**
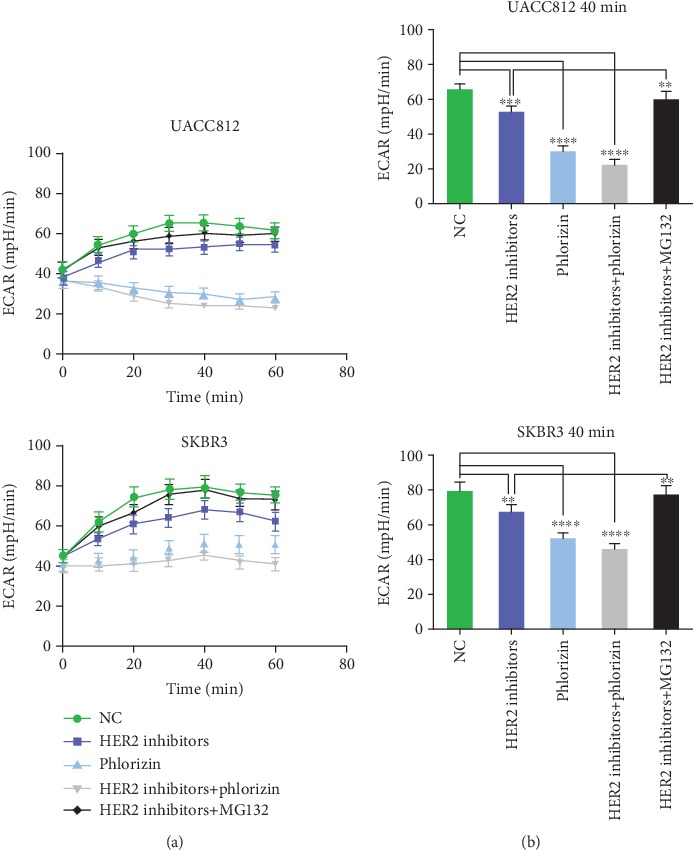
The curve and column results of the ECAR values for UACC812 and SKBR3 cells. (a) The ECAR values of UACC812 and SKBR3 cells treated with 0, 10, 20, 30, 40, 50, and 60 min, respectively. (b) The ECAR values of each group at 40 min in UACC812 and SKBR3 cells. Statistically significant differences between groups were displayed; ^∗∗∗∗^*p* < 0.0001; ^∗∗∗^*p* < 0.001; ^∗∗^*p* < 0.01.

Since SGLT1 plays a vital role in glycolysis, the ECAR, which reflects glycolysis, was measured under different conditions using the Seahorse XF24 Extracellular Flux Analyzer. As shown in Figure 3(a), the ECAR values in both UACC812 and SKBR3 control-treated cells increased significantly with time and reached a maximum at 40 min. The ECAR values of HER2 inhibitor-treated cells were lower than those of control cells, and direct inhibition of SGLT1 with phlorizin reduced the ECAR to a greater extent and had a stable trend overtime. The ECAR values of each treatment at 40 min are shown in Figure 3(b).

### 3.4. HER2 Promotes SGLT1 Stability in a Proteasome-Dependent Manner

Based on many convincing studies that the proteasome acts as a regulator of HER2 protein degradation and signaling, we added a proteasome inhibitor, MG132, to cells treated with HER2 inhibitors to confirm whether this pathway was also involved in the HER2-dependent regulation of SGLT1. As shown in Figure 2, MG132 treatment not only restored HER2 levels but also restored SGLT1 expression in both UACC812 and SKBR3 cells. Consistent with the SGLT1 expression level, the ECAR value in this group also recovered (Figure 3). This suggested that HER2 regulates the expression of SGLT1 in a proteasome-dependent manner.

### 3.5. Knockdown of SGLT1 Inactivates the PI3K/Akt/mTOR Signaling Pathway

**Figure 4:**
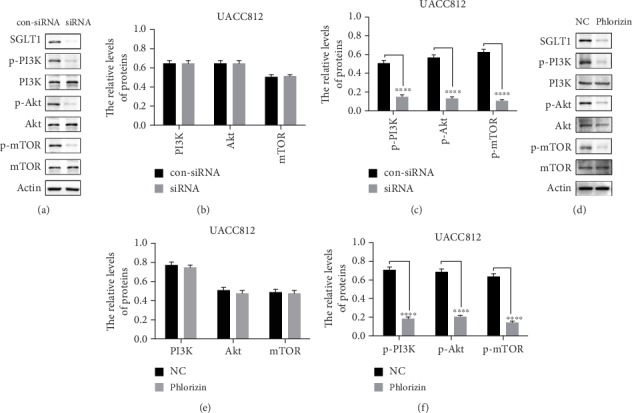
Representative western blot of PI3K/Akt/mTOR pathway-related protein expression levels and quantification relative to actin in UACC812 cells. A representative WB detection result of PI3K/Akt/mTOR pathway-related protein expression levels in (a) SGLT1 siRNA and (d) phlorizin treatment. The relative levels of PI3K/Akt/mTOR protein in (b) SGLT1 siRNA and (e) phlorizin treatment. The relative levels of p-PI3K/p-Akt/p-mTOR protein in (c) SGLT1 siRNA and (f) phlorizin treatment. The results shown were representative of at least three independent experiments; ^∗∗∗∗^*p* < 0.0001.

Previously, SGLT1 has been shown to activate PI3K/Akt/mTOR signaling in other diseases [32]. To verify whether the expression of SGLT1 affected this signal transduction in HER2+ breast cancer cell lines, UACC812 cells were transfected with SGLT1 siRNA and treated with phlorizin, respectively. In Figure 4(a), cells transfected with control siRNA were used as a control. SGLT1 was successfully knocked down in UACC812 cells. In order to characterize the changes in downstream signaling caused by SGLT1 loss, the levels of PI3K/Akt/mTOR signaling pathway proteins were assessed. As expected, compared with control cells, the SGLT1 knockdown cells showed no significant difference in the expression of total PI3K, Akt, or mTOR (Figure 4(b)). However, expression of phosphorylated PI3K, phosphorylated Akt, and phosphorylated mTOR was decreased in the SGLT1 knockdown cells (*p* < 0.0001) (Figure 4(c)). The similar effect can also be seen by phlorizin in Figures 4(d)–4(f). These results suggested that SGLT1 activates the PI3K/Akt/mTOR signaling pathway.

### 3.6. SGLT1 Expression Promotes Proliferation in UACC812 and SKBR3 Cells

**Figure 5:**
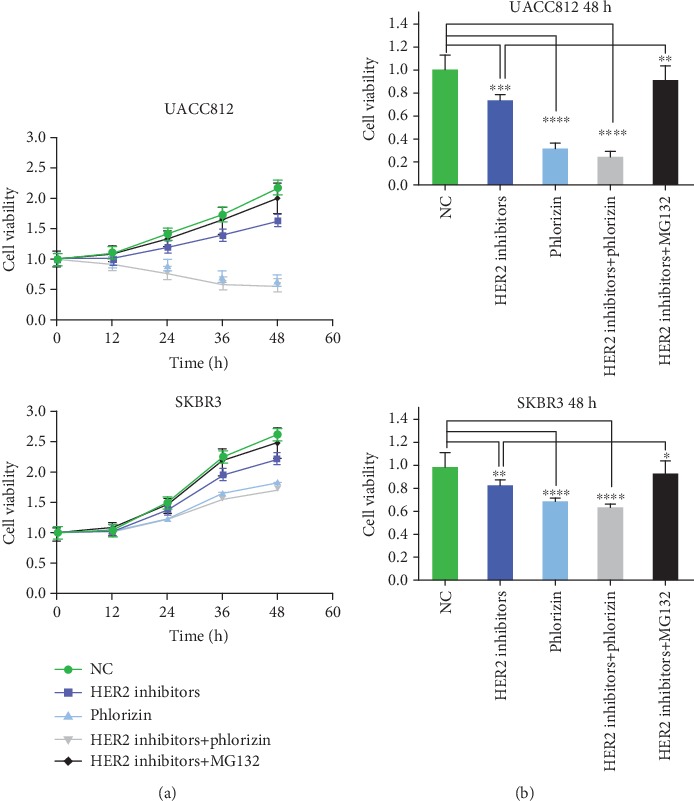
CCK-8 assays for the effect of SGLT1 expression on cell proliferation in UACC812 and SKBR3 cells. (a) The cell viability of UACC812 and SKBR3 cells relative to negative control groups after 0, 12, 24, 36, and 48 h treatment, respectively. (b) The cell viability of each group relative to negative control groups at 48 h in UACC812 and SKBR3 cells. Statistically significant differences between groups were displayed; ^∗∗∗∗^*p* < 0.0001; ^∗∗∗^*p* < 0.001; ^∗∗^*p* < 0.01; ^∗^*p* < 0.05.

Based on the finding that SGLT1 activated PI3K/Akt/mTOR signaling in HER2+ breast cancer, we tested whether SGLT1, stabilized by HER2, promoted cell growth and proliferation. To evaluate the importance of SGLT1 in cell proliferation, CCK-8 assay was performed with the addition of HER2 inhibitors and phlorizin. UACC812 and SKBR3 cells were incubated with different inhibitors for 12 h, 24 h, 36 h, and 48 h. In UACC812 cells, the proliferation rate in cells treated with HER2 inhibitors was lower than that in the control group after 24 h. Treatment with phlorizin alone or with both HER2 inhibitors and phlorizin had a greater inhibitory effect on cell proliferation (Figure 5(a)). The inhibition of cell proliferation was greatest after 48 h (Figure 5(b)). Moreover, the proliferation rate was restored after the addition of MG132, as shown in Figure 5. In SKBR3 cells, the proliferation curves of cells receiving any of the five treatments showed an upward trend (Figure 5), but the proliferation rate of cells treated with HER2 inhibitors was still lower than that of control cells and higher than those of phlorizin- and combination-treated cells after 24 h, 36 h, and 48 h. As with UACC812 cells, MG132 restored the proliferation rate of SKBR3 cells.

### 3.7. SGLT1 Expression Is a Significant Independent Predictor of DFS and OS in Breast Cancer Patients

**Figure 6:**
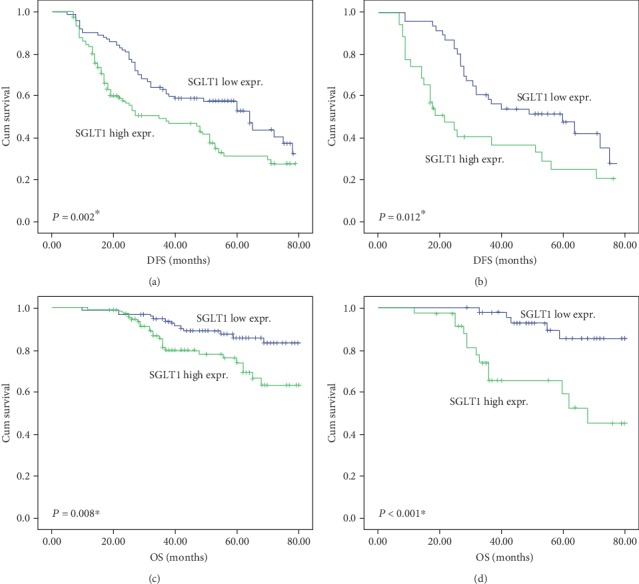
Kaplan-Meier survival analysis according to SGLT1 expression in the whole cohort and HER2+ subtype of breast cancer patients (log-rank test). Association of SGLT1 expression levels with (a) DFS and (c) OS in the whole cohort of breast cancer patients. Association of SGLT1 expression levels with (b) DFS and (d) OS in HER2+ breast cancer patients.

**Figure 7:**
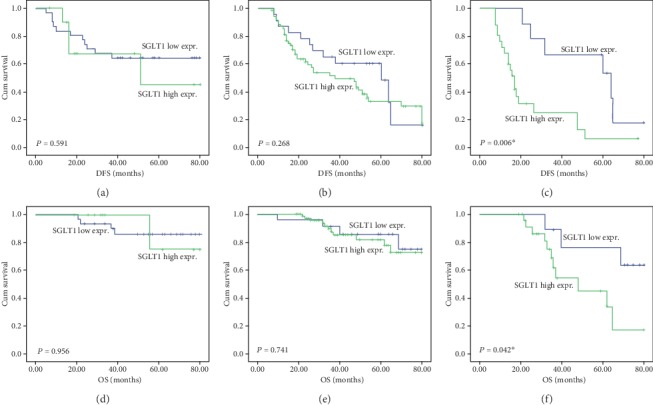
Kaplan-Meier survival analysis according to SGLT1 expression in basal-like, luminal-like, and luminal-HER2+ subtype of breast cancer patients (log-rank test). Association of SGLT1 expression levels with (a) DFS and (d) OS in basal-like breast cancer patients. Association of SGLT1 expression levels with (b) DFS and (e) OS in luminal-like breast cancer patients. Association of SGLT1 expression levels with (c) DFS and (f) OS in LuminalHER2+ breast cancer patients.

Cox's univariate regression analysis was used to evaluate the strength of correlation between clinicopathologic variables of breast cancer patients and survival. Patients with lymph node metastasis and HER2+ status had a shorter DFS (*p* < 0.001 and *p* = 0.001, respectively) and OS (*p* = 0.015 and *p* = 0.001, respectively). As shown in Table 2, high SGLT1 expression was also significantly correlated with shorter DFS (*p* = 0.003) and OS (*p* = 0.011) in the entire patient cohort. These findings were confirmed by Kaplan-Meier estimation. The association of SGLT1 expression with DFS and OS was further elucidated by the survival curves in the whole cohort and when analyzing the different molecular subtypes of breast cancer. In the whole cohort and in patients with the HER2+ subtype, high expression of SGLT1 was associated with poor DFS (*p* = 0.002 and *p* = 0.012, respectively, Figures 6(a) and 6(b)) and OS (*p* = 0.008 and *p* < 0.001, respectively, Figures 6(c) and 6(d)). No prominent associations were observed between SGLT1 expression levels and patient survival in the luminal-like (Figures 7(a) and 7(d)) or basal-like (Figures 7(b) and 7(e)) subtypes. It was noteworthy that high expression of SGLT1 was also correlated with poor DFS (*p* = 0.006) and OS (*p* = 0.042) in patients with the luminal-HER2+ phenotype (Figures 7(c) and 7(f)).

The clinicopathologic features were analyzed for prognostic value using Cox's multivariate regression analysis. As shown in Table 3, SGLT1 and HER2+ status still retained their power to predict shorter DFS (*p* = 0.001 and *p* < 0.001, respectively) and OS (*p* = 0.002 and *p* < 0.001, respectively) in breast cancer patients. Lymph node metastasis remained an independent indicator for DFS (*p* = 0.001), but not for OS (*p* = 0.112).

## 4. Discussion

Breast cancer, the most common malignancy diagnosed in women, is continuously increasing in incidence [34]. It is also characterized by high clinical heterogeneity, which leads to differing prognoses [35, 36]. Although patients with the HER2+ breast cancer subtype are treated by a standard approach, the prognosis remains unsatisfying. The biological behavior of HER2+ breast cancer is extremely complex, and, despite extensive research, the molecular mechanism and signaling pathways involved remain unclear. Consequently, the identification of novel molecular and genetic biomarkers for diagnosis, prognostic estimation, and therapeutic targets for HER2+ breast cancer patients is necessary.

Recent studies have revealed that a series of metabolic alterations, especially in glucose metabolism, allow cancer cells to acquire more nutrients and energy to promote rapid proliferation and metastasis [37, 38]. Previous studies have also reported that SGLT1 is highly expressed in various malignant tumor cells of epithelial origin. SGLT1 sustains stable intracellular glucose levels, regardless of the extracellular glucose concentration [39]. EGFR and HER2 belong to the HER family of receptors and play a role in epidermal growth factor signaling [40]. EGFR is highly expressed in most malignant cells of epithelial origin and plays an important role in the regulation of SGLT1 [41]. A recent study from our collaborators demonstrated that EGFR can stabilize SGLT1 through protein-protein interaction, which prevents proteasomal degradation, independent of the kinase activity of EGFR [19]. Therefore, we continue to conduct a series of in vitro experiments to confirm our hypothesis that SGLT1 can also interact with HER2, thus affecting the prognosis of breast cancer.

Our results confirm that SGLT1 is highly expressed in the HER2+ breast cancer subtype, and overexpression of SGLT1 is significantly associated with lymph node metastasis and HER2+ status. In vitro studies also confirm the high expression of SGLT1 in HER2+ breast cancer cell lines, and inhibition of HER2 decreases the expression of SGLT1. Further, the ECAR, which indirectly reflects glycolysis, is also affected by HER2 or SGLT1 inhibition. The ECAR pattern reflected the SGLT1 expression pattern. In addition, we determined that MG132 restores SGLT1 expression, indicating that the proteasome is responsible for the degradation of SGLT1. MG132 treatment also restored the ECAR in treated cells. To better understand the role of SGLT1 in HER2+ breast cancer, SGLT1 was knocked down in UACC812 and SKBR3 cells. This abrogated PI3K/Akt/mTOR signaling and significantly decreased the proliferative ability. Thus, high expression of SGLT1 could guarantee the ability of cancer cells to uptake enough glucose for ATP generation via anaerobic glycolysis. Hence, enhanced expression of SGLT1 may at least be partially responsible for the progression of HER2+ breast cancer. Finally, the prognostic capability of SGLT1 expression in breast cancer subgroups was also assessed. High expression of SGLT1 is significantly associated with shorter survival in the whole cohort in both univariate and multivariate analyses, as well as in HER2+ and luminal-HER2+ breast cancer patients. Therefore, SGLT1 is an independent adverse prognostic factor in breast cancer, especially in HER2+ breast cancer. In light of these results, inhibition or downregulation of SGLT1 is considered to be a promising strategy to prevent tumor progression and may be combined with cytostatic drugs.

In summary, we examined SGLT1 expression in both breast cancer tissues and cell lines and verified that SGLT1 promotes cell proliferation by activation of PI3K/Akt/mTOR signaling in HER2+ breast cancer. We also related the levels of SGLT1 to the prognosis of breast cancer, especially the HER2+ subtype, and demonstrated that high expression of SGLT1 is an independent and reliable biomarker for the prediction of patient survival. SGLT1 may indicate a promising therapeutic target for breast cancer treatment.

## Figures and Tables

**Table 1:** Associations between SGLT1 protein expression levels and clinicopathologic variables of the breast cancer patient cohort.

Variables	SGLT1 protein All cases (*n* = 216^a^)	Negative expression	Positive expression	*p* value
Age				
≤50	131	62	69	0.706
>50	85	38	47	
Tumor size (cm)				
≤2	103	42	61	0.120
>2	113	58	55	
Lymph node status				
Negative	120	66	54	**0.004**
Positive	96	34	62	
Menopause status				
Premenopausal	112	48	64	0.293
Postmenopausal	104	52	52	
ER status				
Negative	160	78	82	0.222
Positive	56	22	34	
PR status				
Negative	159	79	80	0.150
Positive	57	22	35	
HER2 status				
Negative	91	35	56	**0.049**
Positive	125	65	60	

Significant *p* values (*p* < 0.05) are indicated in bold. ^a^Number of patients.

**Table 2:** Cox's univariate regression analysis of the patient survival in breast cancer with respect to clinical parameters and cancer biological factors.

Variables	*n* ^a^	DFS HR (95% CI)^b^	*p*	OS HR (95% CI)^b^	*p*
Age (years)			0.508		0.747
≤50	131	1		1	
>50	85	1.131 (0.786-1.627)		0.900 (0.475-1.706)	
Tumor size (cm)			0.680		0.164
≤2	103	1		1	
>2	113	1.079 (0.751-1.552)		1.627 (0.819-3.232)	
Lymph node status			**<0.001**		**0.015**
Negative	120	1		1	
Positive	96	2.125 (1.483-3.045)		2.248 (1.170-4.318)	
Menopausal			0.299		0.358
Premenopausal	112	1		1	
Postmenopausal	104	0.828 (0.580-1.182)		0.741 (0.390-1.404)	
ER status			0.714		0.237
Negative	160	1		1	
Positive	56	0.927 (0.616-1.393)		0.610 (0.268-1.385)	
PR status			0.703		0.780
Negative	159	1		1	
Positive	57	0.924 (0.615-1.389)		0.902 (0.438-1.858)	
HER2 status			**0.001**		**0.001**
Negative	91	1		1	
Positive	125	1.925 (1.307-2.835)		4.142 (1.731-9.909)	
SGLT1			**0.003**		**0.011**
Negative	100	1		1	
Positive	116	1.738 (1.208-2.500)		2.402 (1.225-4.711)	

Significant *p* values (*p* < 0.05) are indicated in bold. ^a^Number of patients. ^b^Hazard ratio (HR) (CI confidence interval) of Cox's univariate regression analysis.

**Table 3:** Cox's multivariate regression analysis of the patient survival in breast cancer with respect to clinical parameters and cancer biological factors.

Variables	*n* ^a^	DFS HR (95% CI)^b^	*p*	OS HR (95% CI)^b^	*p*
Lymph node status			**0.001**		0.112
Negative	120	1		1	
Positive	96	1.863 (1.291-2.687)		1.712 (0.882-3.323)	
HER2 status			**<0.001**		**<0.001**
Negative	91	1		1	
Positive	125	2.133 (1.432-3.178)		4.959 (2.046-12.020)	
SGLT1			**0.001**		**0.002**
Low	100	1		1	
High	116	1.891 (1.297-2.756)		2.935 (1.469-5.865)	

Significant *p* values (*p* < 0.05) are indicated in bold. ^a^Number of patients. ^b^Hazard ratio (HR) (CI confidence interval) of Cox's multivariate regression analysis.

## Data Availability

The data used to support the findings of this study are available from the corresponding author upon request.

## Abbreviations

DFS: Disease-free survival
ECAR: Extracellular acidification rate
EGFR: Epidermal growth factor receptor
ER: Estrogen receptor
HER: Human epidermal growth factor receptor
IGF-1R: Insulin growth factor-1 receptor
IHC: Immunohistochemistry
mTOR: Mammalian target of rapamycin
OS: Overall survival
PI3K: Phosphoinositide 3-kinase
PR: Progesterone receptor
SGLT: Sodium-dependent glucose transporter.
